# Glutathione-conjugating and membrane-remodeling activity of GDAP1 relies on amphipathic C-terminal domain

**DOI:** 10.1038/srep36930

**Published:** 2016-11-14

**Authors:** Nina Huber, Christoph Bieniossek, Konstanze Marion Wagner, Hans-Peter Elsässer, Ueli Suter, Imre Berger, Axel Niemann

**Affiliations:** 1 Institute of Molecular Health Sciences, Department of Biology, ETH Zürich, Swiss Federal Institute of Technology, 8093 Zurich, Switzerland; 2 Department of Psychiatry and Behavioral Sciences, Stanford University School of Medicine, Stanford, CA, USA; 3 European Molecular Biology Laboratory, Grenoble Outstation, 38042 Grenoble, France; 4 Roche Pharma Research and Early Development, Infectious Diseases Discovery, Roche Innovation Center Basel, F. Hoffmann-La Roche Ltd, Grenzacherstrasse 124, 4070 Basel, Switzerland; 5 Department of Cytobiology and Cytopathobiology, Philipps University of Marburg, 35033 Marburg, Germany; 6 School of Biochemistry, Bristol University, Bristol BS8 1TD, United Kingdom

## Abstract

Mutations in the ganglioside-induced differentiation associated protein 1 (GDAP1) cause severe peripheral motor and sensory neuropathies called Charcot-Marie-Tooth disease. GDAP1 expression induces fission of mitochondria and peroxisomes by a currently elusive mechanism, while disease causing mutations in *GDAP1* impede the protein’s role in mitochondrial dynamics. *In silico* analysis reveals sequence similarities of GDAP1 to glutathione *S*-transferases (GSTs). However, a proof of GST activity and its possible impact on membrane dynamics are lacking to date. Using recombinant protein, we demonstrate for the first time theta-class-like GST activity for GDAP1, and it’s activity being regulated by the C-terminal hydrophobic domain 1 (HD1) of GDAP1 in an autoinhibitory manner. Moreover, we show that the HD1 amphipathic pattern is required to induce membrane dynamics by GDAP1. As both, fission and GST activities of GDAP1, are critically dependent on HD1, we propose that GDAP1 undergoes a molecular switch, turning from a pro-fission active to an auto-inhibited inactive conformation.

Ganglioside-induced differentiation associated protein 1 (GDAP1) is an integral, tail-anchored protein of the mitochondrial outer membrane (MOM) and the peroxisomal membrane^1^,^2^,^3^. GDAP1 is predominantly expressed in neural cells where it mediates mitochondrial and peroxisomal fragmentation dependent on ubiquitously expressed fission factors (Fis1, Drp1, and Mff)^1^,^2^,^3^,^4^,^5^. Mutations in *GDAP1* are associated with the hereditary motor and sensory neuropathy Charcot-Marie-Tooth (CMT) disease^6^,^7^,^8^. *In vitro* studies revealed that recessively inherited mutant forms of GDAP1 (rmGDAP1) exhibit reduced fission-promoting activity, whereas dominantly inherited mutant forms (dmGDAP1) interfere with mitochondrial fusion^4^,^9^. Furthermore, while GDAP1 expression is protective in glutamate-induced toxicity, this protection is reduced in rmGDAP1s, dependent on their residual fission-capacity^10^.

*In silico* analyses predict that GDAP1 is a glutathione *S*-transferase (GST)^11^,^12^,^13^. GSTs are multifunctional proteins involved in cellular detoxification or regulating cellular glutathione (GSH) levels, hormone biosynthesis, and intracellular signaling^14^,^15^. These enzymes are classified according to their protein sequence and structure, dimerization motif, features of the catalytic active centre, and substrate specificity^14^,^15^,^16^. GDAP1 is an integral membrane protein and contains in addition to its transmembrane domain (TMD) a further C-terminal hydrophobic domain (HD1). Two domains in GDAP1 (GST-N and GST-C) show similarities to cytosolic zeta-, omega-, or theta-class GSTs^11^,^13^. Between its putative GST-N and GST-C domains, GDAP1 contains an extended interdomain linker which may adopt two additional alpha-helices^13^. These features are also present in the GDAP1-paralogue GDAP1like1, possibly constituting a new class of GST proteins^13^. In response to changes in the cellular redox state, the cytosolic GDAP1like1 translocates to mitochondria, integrates into the MOM and causes mitochondrial fission^17^. This translocation and integration of GDAP1like1 into the MOM is specifically caused by an increase in the concentration of the oxidized form of glutathione *in vitro* and *in vivo* where it is capable of substituting for the loss of GDAP1 in the central nervous system of GDAP1-deficient mice^17^. Interestingly, mitochondrial translocation from the cytosol under oxidative stress conditions has previously been described also for other GSTs such as GSTA4–4 and GSTPi^18^,^19^,^20^,^21^. However, in spite of considerable effort, attempts to demonstrate GST activity of GDAP1 and the functional consequences have not met success to date^5^,^11^,^12^,^13^.

We show here that highly purified recombinant human GDAP1 is indeed a GST enzyme, and we demonstrate specific GSH-conjugating activity *in vitro*. We discovered that GST activity is regulated by the hydrophobic domain 1 (HD1), which exerts an autoinhibitory function. HD1 could adopt an amphipathic pattern and this amphipathic pattern is necessary to induce remodelling of organelles-mimicking liposomes by Gdap1. We propose a model of action with two different, interconvertible conformations of GDAP1: a GST active state with an exposed HD1 mediating mitochondrial and peroxisomal fission, and a GST-inactive state caused by an autoinhibitory binding mode of HD1.

## Results

### GDAP1 forms dimers

**Figure 1:**
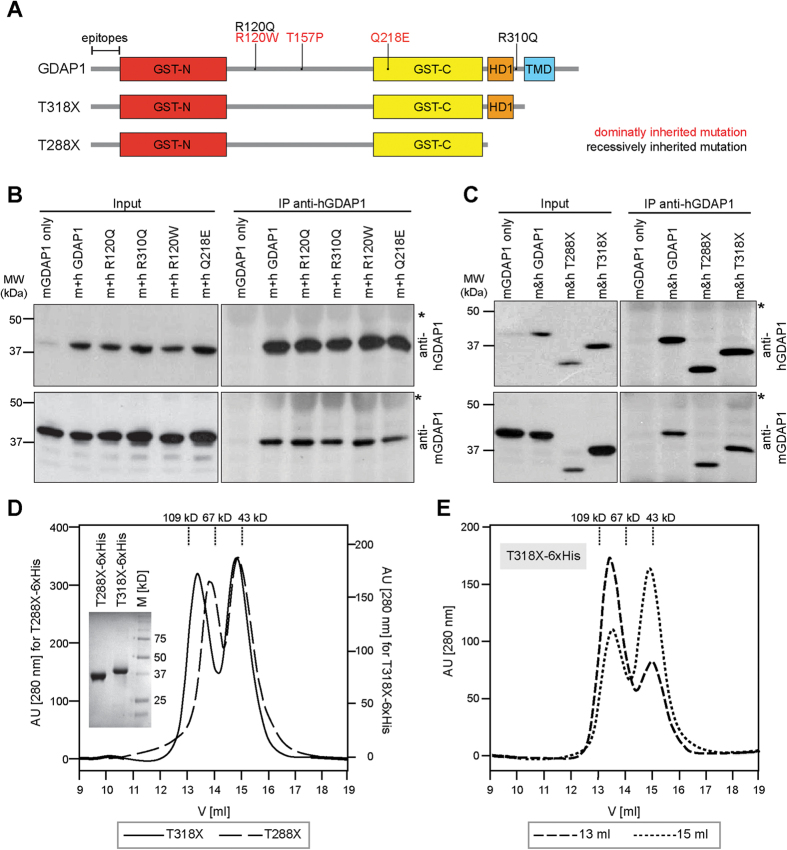
GDAP1 forms homodimers. (**A**) Schematic representation of the domain structure of GDAP1 (conserved GST domains: GST-N and GST-C; hydrophobic domain 1: HD1, transmembrane domain: TMD). The localization of disease-associated missense mutations used in this study and GDAP1 truncations lacking the TMD (GDAP1 T318X) or TMD and HD1 (GDAP1 T288X) are illustrated. Human and mouse-specific antisera were generated against species-specific epitopes of the N-terminal region (epitopes). (**B**,**C**) Immunoprecipitation performed with the human-specific anti-GDAP1 antibody co-precipitates the corresponding mouse isoforms from co-transfected HEK-293T cells. Without human GDAP1 co-expression, the murine GDAP1 is not precipitated. *Signal of IgG heavy chains. (**D**) S200 elution profiles and a Coomassie blue stained SDS-gel of the purified proteins. Peak fractions of T318X-6xHis were pooled separately and re-chromatographed under identical conditions (**E**) consistently resulting in 2 peaks.

GST enzymes critically depend on dimerization in order to be catalytically active^14^. To test the dimerization capacity of GDAP1, we transiently co-expressed human and mouse isoforms of full-length and untagged GDAP1 or disease-causing mutant forms of GDAP1 (Fig. 1A) in HEK-293T cells and performed co-immunoprecipitation (co-IP) experiments using human-isoform-specific anti-GDAP1 antibodies. IPs of human GDAP1 co-precipitated mouse Gdap1 (Fig. 1B). In addition, all tested rmGDAP1 (R120Q, R310Q) and dmGDAP1 (R120W, Q218E) were also able to form homodimers (Fig. 1B). Consistently, human GDAP1 was co-precipitated with the murine specific Gdap1 antibody (see Supplementary Fig. S1A), confirming that GDAP1 wildtype or disease mutants can dimerize.

We next tested whether the C-terminal hydrophobic domains of GDAP1 are dispensable for dimerization. GDAP1 lacking the TMD (GDAP1 T318X) or lacking the TMD and HD1 (GDAP1 T288X) were co-expressed as human and murine truncated proteins in HEK-293T cells (Fig. 1A). Co-immunoprecipitation revealed that both truncated forms homo-dimerize (Fig. 1C, see Supplementary Fig. S1B). These data are consistent with previous experiments using truncated or tagged GDAP1 constructs^11^,^22^. We conclude that GDAP1 T288X and GDAP1 T318X are capable of dimerization, which is a pre-requisite for catalytic activity.

### GDAP1 is catalytically active and mediates glutathione-conjugation *in vitro*

Recombinant, full-length or truncated GDAP1 expressed in bacteria is not soluble (data not shown), consistent with previous reports^11^. We therefore tested eukaryotic expression using MultiBac, a baculovirus-based insect cell protein expression system^23^. We expressed two soluble His-tagged GDAP1 variants (GDAP1 T288X-6xHis and GDAP1 T318X-6xHis) in large amounts. The proteins were purified to homogeneity by metal affinity chromatography followed by size-exclusion chromatography (SEC) (Fig. 1D). Elution profiles showed two peaks, indicating that both GDAP1 variants exist in a monomeric and a dimeric form, validating our co-immunoprecipitation experiments (Fig. 1E). Re-chromatographing either the monomeric or dimeric peak fractions by SEC resulted again in two peaks, consistent with a dynamic monomer-dimer equilibrium (Fig. 1E).

We assayed GST activity with the highly purified GDAP1 proteins using different model substrates. Our experiments revealed GSH-conjugating activity for GDAP1 T288X to ethacrynic acid, *p*-Nitrobenzylchloride, and 1,2-Epoxy-3-(4-nitrophenoxy)propane, but not to the classic GST-substrate 1-Chloro-2,4-dinitrobenzene (Table 1). In addition, both GDAP1 variants failed to bind GSH-sepharose (not shown). This activity profile is characteristic for theta-class GSTs^16^. Our results thus unequivocally demonstrate that GDAP1 has theta-class-like GST activity *in vitro*^11^,^13^,^16^.

### HD1 regulates GDAP1 GST- activity and mediates membrane curvature *in vitro*

**Figure 2:**
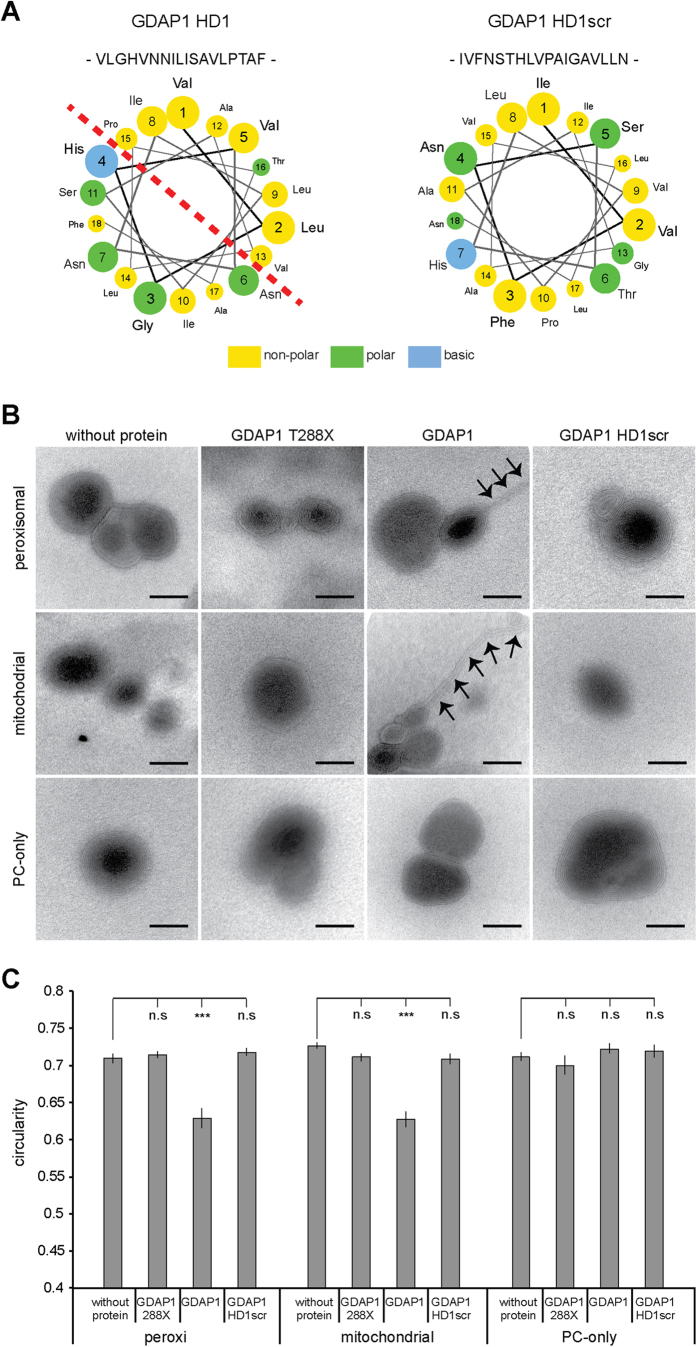
GDAP1 tubulates liposomes dependent on the HD1 and the lipid composition. (**A**) Helical wheel representation of residues of the HD1 reveals the amphipathic pattern of hydrophilic and hydrophobic amino acids. This pattern is broken up by scrambling the primary sequence of the HD1 (HD1scr). (**B**) Electron microscopy of negatively stained liposomes of phosphatidylcholine (PC) or of lipid compositions resembling the mitochondrial outer membrane (Mito) or peroxisomal membrane (Peroxi) were incubated with *in vitro* translated GDAP1, GDAP1 HD1scr, GDAP1 lacking HD1 and TMD (GDAP1 T288X), or were left untreated. All liposomes have primarily multilamellar appearances. Only the addition of GDAP1 to Mito- and Peroxi-liposomes caused tubulation (arrows). Scale bars: 50 nm. (**C**) Per preparation 16 to 25 electron micrographs were taken blindly. On electronic pictures all discrete liposomes were selected automatically and the circularity of the objects was determined. The graph depicts the mean and the s.e.m. of all liposomes per condition (n = 79 to 197) from independent preparations, paired t-test ***P-value < 0.0005.

Strikingly, in contrast to GDAP1 T288X-6xHis, the longer construct GDAP1 T318X-6xHis containing the C-terminal HD1, did not display detectable GST activity with any of the substrates tested (Table 1). These results suggest a regulatory function of HD1 acting as an autoinhibitory domain, possibly by locking the protein in an inactive conformation. Interestingly, HD1 is essential to execute GDAP1-induced peroxisomal and mitochondrial fission^2^,^3^. Computational protein sequence analysis revealed a high propensity for HD1 to adopt an amphipathic helix, where polar and hydrophobic residues disproportionate to two distinct halves of the structure (Fig. 2A). Amphipathic helices with a similar distribution of polar and apolar amino acids are known to interact with membranes and induce membrane bending, curvature, and remodelling^24^,^25^. Recombinant full-length GDAP1 turned out to be not soluble when expressed in either bacteria^11^ or even in insect cells (data not shown). However, we have previously demonstrated that *in vitro* translated full-length GDAP1 integrates readily into liposomes^2^. We now made use of this experimental approach to test the amphipathic characteristics of GDAP1’s HD1. We incubated *in vitro* translated GDAP1 with liposomes of different compositions^26^ and analyzed the liposome morphology by negative staining (Fig. 2B). Liposomes without any addition of *in vitro* translated protein have a round shape, which was quantified by morphometric analysis (Fig. 2C). The addition of GDAP1 T288X lacking both HD1 and TMD served as negative control as this protein is not targeted to membranes^2^,^3^. In contrast to the control, *in vitro* translated full-length GDAP1 added to liposomes mimicking outer mitochondrial or peroxisomal membranes induced membrane outfoldings and significantly deformed the liposomes, while pure phosphatidylcholine liposomes are not deformed by the addition of the same translation mix (Fig. 2B,C). To investigate the role of HD1’s amphipathic pattern for this membrane remodelling activity of GDAP1, we also translated *in vitro* full-length GDAP1 with a scrambled HD1 (HD1scr) to break the amphipathic structure (Fig. 2A). Similar to the negative-control, HD1scr did not deform the liposome surface of all three membrane-compositions (Fig. 2B,C).

## Discussion

We provide here first experimental evidence that GDAP1 has a GSH-conjugation activity typical for theta-class GSTs. While full-length GDAP1 cannot be purified in soluble form from *E. coli* or insect cell (not shown), C-terminal truncated recombinant GDAP1 proteins without the C-terminal transmembrane domain (TMD) are readily expressed and soluble. More interestingly, we found that only GDAP1 lacking both its C-terminal TMD and HD1 has GSH-conjugating activity. By size-exclusion chromatography (SEC) and immunoprecipitation experiments we confirm that GDAP1 with HD1 but without the TMD also forms dimers. We conclude that loss of GST-activity in the presence of HD1 is not caused by a protein dimerization deficit and hypothesize that HD1 might have an auto-inhibitory function in regulating the enzyme activity of GDAP1.

To understand the potential role of HD1 in GDAP1 we performed *in silico* analysis, which predicted an amphipathic pattern for HD1, a structure known for its membrane-remodeling activities. Indeed, the addition of *in vitro* translated full-length GDAP1 to liposomes mimicking the peroxisomal or outer mitochondrial membrane composition lead to a deformation of these liposomes. Incubation oft the same liposomes with GDAP1 with a scrambled HD1 sequence or GDAP1 lacking the TMD does not alter the liposomal shape. This corroborates our previous findings showing that the HD1 is essential to mediate GDAP1-induced mitochondrial and peroxisomal fission^3^,^4^. However, we could not link the membrane remodeling capacity of GDAP1 with GDAP1’s GST activity as the *in vitro* translated full-length GDAP1 did not prove to have GST activity (not shown) and the GST-active recombinant GDAP1 is not targeted to membranes as it lacks the tail-anchor domain^2^.

**Figure 3:**
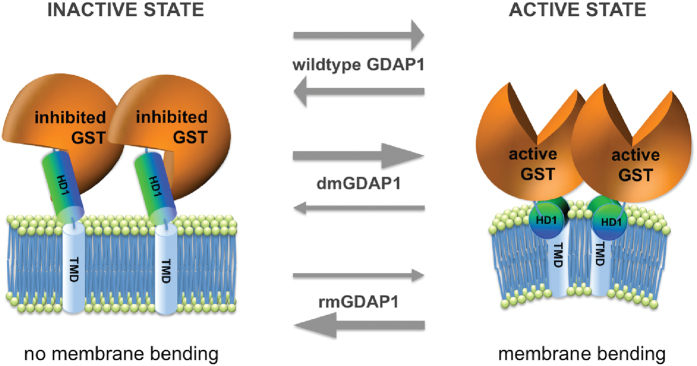
Two-conformation molecular switch model of GDAP1: Active and inactive states. The fission factor GDAP1 is anchored with its C-terminal TMD into the mitochondrial outer membrane or peroxisomal membrane. Its N-terminal GST-domains and the HD1 are cytosolic. We hypothesize that GDAP1’s fission activity is regulated by its enzymatically active GST-portion and dependent on HD1. In the inactive state GDAP1 is not capable of inducing fission. Instead, HD1 is blocking the GST activity in an autoinhibitory fashion (left). In the active state, GDAP1 induces fission (right) dependent on the amphipathic pattern of HD1, which induces membrane curvature. Wild type GDAP1 acts as a molecular switch between the active and the inactive state, depending on specific stimuli. dmGDAP1 preferentially adopt the active conformation resulting in toxic hyper-fission activity, whereas rmGDAP1 have lost or markedly reduced fission activity as they preferentially adopt the inactive state. Experimental evidence for the different states is currently lacking due to the absence of high-resolution structural data.

Implementing our new findings together with results from previous reports^2^,^3^,^4^,^10^,^11^ into a hypothetical working model, we suggest that GDAP1 function may be mechanistically regulated by adopting two different conformations, an active and inactive state (Fig. 3). In the active state, GST activity is not inhibited, owing to the fact that HD1 is exposed and dips into the cytosolic leaflet of the MOM or peroxisomal membrane to mediate membrane curvature. In contrast, in the inactive state the interaction with the membrane is lost and HD1 acts as an autoinhibitory domain locking the N-terminal cytosolic domains of GDAP1 in a fission- and GST-inactive conformation. We propose that GDAP1 switches between these distinct molecular conformations.

In the absence of high-resolution atomic structures of GDAP1, experimental prove for our proposed model is currently lacking. Notwithstanding, evidence exists that is consistent with our model, including the observed properties of point mutated disease-associated forms of GDAP1. Recessively inherited mutations cluster within the coding region of the N-terminal, cytosolic GST-N, GST-C, and interdomain of *GDAP1*^6^, suggesting that the function of GDAP1’s GST domain is impaired in recessively inherited mutant forms. We speculate that GDAP1 expression may be protective by its GST activity, and GSTs were proposed to exert such functions^18^,^19^,^20^. However, our attempts to determine GST activity of disease-associated mutants failed, as all tested recombinant mutated GDAP1 proteins isolated from insect cells were not soluble, indicating non-functional folding of the mutants.

Expressing rmGDAP1 is associated with a loss or markedly reduced fission capacity, while dmGDAP1 still induce fission but block or delay mitochondrial fusion^1^,^5^. Based on our model we speculate that mutant forms of GDAP1 associated with CMT are likely to favour one of the two protein conformations. dmGDAP1 protein might be in a pro-active, fission-inducing conformation, resulting in a fragmented mitochondrial network, whereas the rmGDAP1 remain in the inactive, fission- and GST-inhibited conformation^4^,^9^. This suggests that GDAP1 function relies on an activating stimulus to switch between conformations. We have previously described a similar activation switch for the cytosolic GDAP1-paralogue GDAP1like1, which translocates to mitochondria and integrates into the MOM upon changes of the cellular redox conditions, inducing mitochondrial fission^17^. In analogy we favour the interpretation that GDAP1’s GST domains function as a redox sensor, rather than a *bona fide* GST enzyme, changing its conformational state upon activating stimuli, which regulates the release of the autoinhibitory HD1 domain to execute fission by interaction with the organelle’s outer leaflet.

Besides the induction of mitochondrial and peroxisomal fission, GDAP1 has been linked to the regulation of intracellular Ca(2+) homeostasis^27^,^28^. Interestingly, loss of GDAP1 expression can be modified by junctophilin-1, which restores the store-operated Ca(2+) entry (SOCE)^27^. Although the morphological changes of mitochondria are not sufficient to explain changes in intracellular Ca(2+) homeostasis^29^, intracellular Ca(2+) levels and redox conditions are very tightly linked^30^. GDAP1 might function as a mitochondrial redox sensor and transducer of ROS signals to SOCE. Similarly, mitochondrial fusion factor mitofusin-2 (MFN2), which is mutated in axonal forms of CMT (OMIM: 609260), influences the entry of Ca(2+) when mitochondria are depolarized^31^. Furthermore, oxidized glutathione levels directly influence MFN2 fusion activity^32^. Taken together these results point to a common theme in CMT caused by mutations in *GDAP1* or *MFN2*, which converge on sensing of redox stress conditions and result in the regulation of mitochondrial morphology and intracellular Ca(2+) levels. However the precise conditions stimulating these proteins *in vivo* and the resulting molecular and cellular reactions remain to be elucidated.

## Materials and Methods

### Cell culture

HEK-293T cells were cultured and transfected with Lipofectamine 2000 (Invitrogen) as described previously^1^,^3^. The insect cells Sf21 were cultured, maintained and infected as described^23^.

### Plasmids and antibodies

Human GDAP1 (hGDAP1) missense mutations and truncations were cloned into the pcDNA3.1 expression vector as described previously^1^,^2^. The constructs GDAP1 T288X and GDAP1 T3188X and their mutations were cloned into vector pFL from the MultiBac system. All constructs comprise a hexa-histidine tag at their C-terminus^23^. For Western blotting and immunocytochemistry, anti-mouse GDAP1 (Pineda, Berlin, Germany) and anti-human GDAP1 (Pineda, Berlin, Germany) were used as described^1^,^9^.

### Recombinant protein production

Proteins were expressed as described in ref. 23. The constructs were all purified in the same 2-step manner. Cells were solubilized in buffer A (25 mM Tris pH8, 200 mM NaCl) and lyzed by freeze-thaw. Soluble and insoluble fractions were separated by ultra-centrifugation (1 h at 45 kRPM in Beckman Ti70 rotor). The supernatant was then loaded to a pre-equilibrated Talon affinity column (Clontech, Stain-Germain-en-Laye, France) and eluted with a gradient changing to buffer B (25 mM Tris pH8, 200 mM NaCl, 250 mM imidazole). The relevant fractions were pooled and supplemented with 2 mM DTT. Finally, the concentrated fractions were purified with a pre-equilibrated Superdex 200 10/300 (GE Healthcare, Uppsala, Sweden) in buffer C (20 mM Tris pH 8.0, 100 mM NaCl, 1 mM DTT, 1 mM EDTA). The *in vitro* translation of GDAP1 wildtype and mutant forms was performed using the TNT T7 Quick Coupled reticulocyte system (Promega, Madison, USA) as described previously^2^.

### Immunoprecipitation

30 μl wet Protein A-Sepharose beads (GE Healthcare, Uppsala, Sweden) were incubated overnight with 5 μl GDAP1 antibody serum. Transfected HEK-293T were harvested in lysis buffer (50 mM Tris pH 7.5, 150 mM NaCl, 1 mM EDTA, 1% Triton X-100, 1:100 Protease inhibitor cocktail (Sigma, St. Louis, USA)), centrifuged for 5 min at 800 g and the cleared supernatant was than incubated with the antibody coupled beads for 2 h. Beads were washed four times with lysis buffer, and subsequently the samples were boiled at 95 °C in SDS-loading buffer and subjected to immunoblotting analysis.

### GST activity measurements

For all GST activity assays 50 μg of recombinant protein (in buffer 20 mM Tris pH 8.0, 100 mM NaCl, 1 mM DTT, 1 mM EDTA) in a total reaction volume of 200 μl using the conditions listed in Supplementary Table 1 (see Supplementary Table 1) were used^33^,^34^. Crude rat liver extract served as positive control and buffer only as negative control for any assay. The absorbance was measured every 30 sec in a multi-well plate Photometer at room temperature for ten to twenty minutes (SpectraMax190, Molecular Devices, Sweden).

### Liposome generation and analysis

Liposomes were prepared using 5 mM stock solutions in methanol:chloroform (1:4) of phosphtidycholine (PC, Sigma, St. Louis, USA), L-α-phosphatidylethanolamine (PE), 1,2-dioleoyl-sn-glycero-3-phospho-L-serine (18:1; PS), L-α-phosphatidylinositol (PI), cardiolipin (CA). Lipids were purchased from Avanti Polar Lipids Inc., if not indicated differently. To prepare liposomes in the desired composition, 20 μl lipid-mixture (PC = 20 μl PC; Peroxi = 11 μl PC, 6 μl PE, 1 μl PS, 1 μl PI, 1 μl CL^26^; Mito = 10.8 μl PC, 4 μl PE, 5.2 μl CL^35^ were dried under vacuum for 2 h. Lipids were resuspended in 400 μl buffer (10 mM HEPES pH 7.4, 1 mM EDTA, 250 mM sucrose) and passed ten times through two 100-nm pore size polycarbonate filters (Avestin). 50 μl liposome solution were incubated with 10 μl *in vitro* translate or were left untreated for 4 hours on ice, mixed 1:1 with 2% phosphotungstic acid (pH 7.2) and immediately placed on Formvar-coated nickel grids (200 mesh, PLANO) for 4 minutes. The liquid was removed and the grids were dried for 2 hours under vacuum and examined in blinded fashion with a Zeiss EM 109S electron microscope (Zeiss, Oberkochen, Germany). On the electronic pictures all separate liposomes were selected with the magic wand tool in Adobe Photoshop and the circularity of the objects was determined.

### Software

Data analysis was performed using Excel (Microsoft) and the amphipathic pattern was determined by the modeling software http://cti.itc.virginia.edu/~cmg/wheel/wheelApp.html.

## Additional Information

**How to cite this article**: Huber, N. *et al*. Glutathione-conjugating and membrane-remodeling activity of GDAP1 relies on amphipathic C-terminal domain. *Sci. Rep*. **6**, 36930; doi: 10.1038/srep36930 (2016).

**Publisher’s note**: Springer Nature remains neutral with regard to jurisdictional claims in published maps and institutional affiliations.

## Supplementary Material

Supplementary Information

## Figures and Tables

**Table 1:** GST-activities of GDAP1 T288X and GDAP1 T318X with model substrates.

Substrate	Specific activity (nmol/min per mg protein)	
	GDAP1 T288X	GDAP1 T318X
1-Chloro-2,4-dinitrobenzene (CDNB)	11 ± 4 (12)	n.d. (10)
Cumene hydroperoxide	n.d. (18)	n.d. (12)
*t*-Buthyl hydroperoxide	n.d. (8)	n.d. (8)
2-Hydroxyethylsulphide	n.d. (8)	n.d. (8)
Ethacrynic acid (EA)	26 ± 2 (8)	n.d. (8)
*p*-Nitrobenzylchloride (*p*NBC)	96 ± 3 (8)	n.d. (8)
*p*-Nitrophenylacetate (*p*NPA)	n.d. (8)	n.d. (8)
7-Chloro-4-nitrobenzeno-2-oxa-1,3-diazol	n.d. (8)	n.d. (8)
1,2-Epoxy-3-(4-nitrophenoxy) propane (EPNP)	820 ± 116 (10)	n.d. (8)

*Data are shown as means* ± *standard deviation; n.d. indicates where the specific activity was* <*10. The number of assays per substrate* (*n*) *is shown in brackets behind the specific activities*.
