# Supplementary material for: Glutathione-conjugating and membrane-remodeling activity of GDAP1 relies on amphipathic C-terminal domain
Source: Sci Rep. 2016 Nov 14;6:36930. doi: 10.1038/srep36930 (PMC5107993; doi:10.1038/srep36930)
Supplement: Supplementary Information [file srep36930-s1.pdf]

Glutathione-conjugating and membrane-remodeling activity of GDAP1 relies on amphipathic C-terminal domain

Nina Huber, Christoph Bieniossek, Konstanze Marion Wagner, Hans-Peter Elsässer, Ueli Suter, Imre Berger, and Axel Niemann

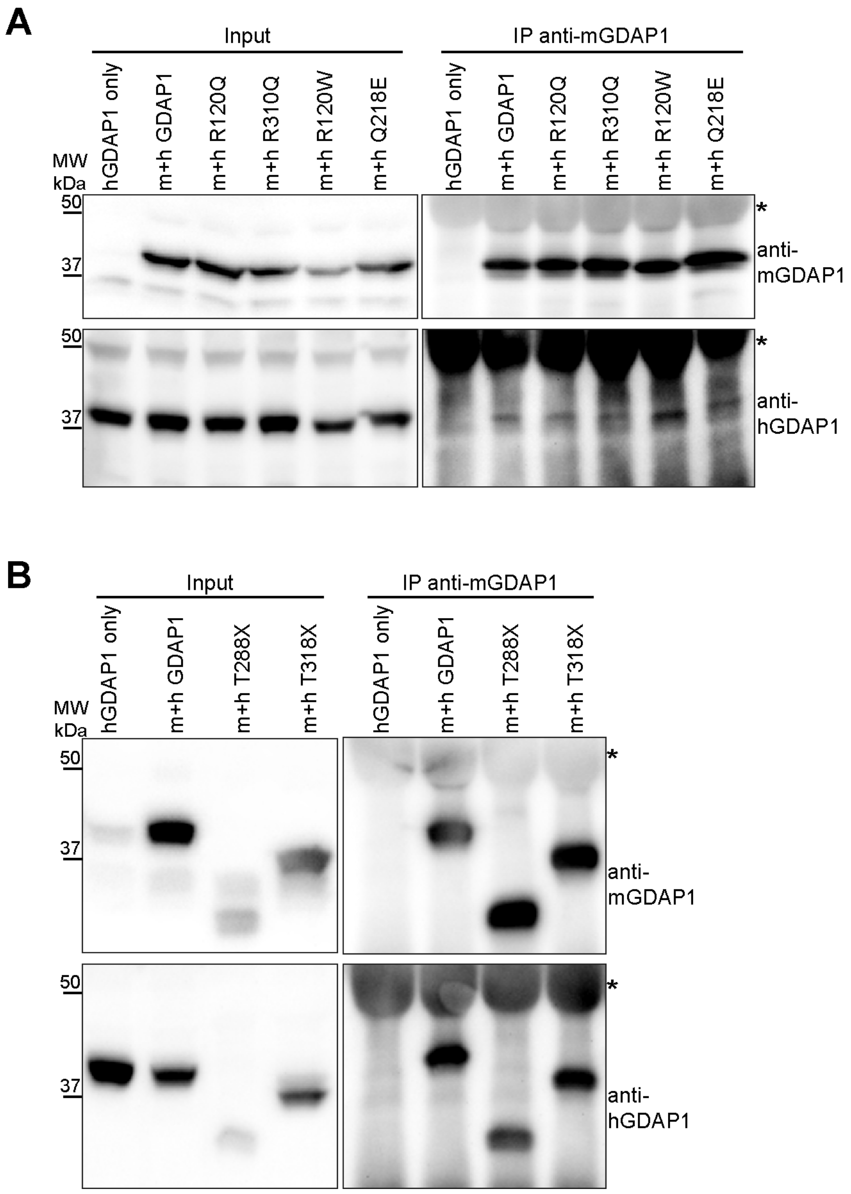

Supplementary Figure S1: CMT-associated mutant forms of GDAP1 do not impair dimerization. (A, B) Immunoprecipitations performed with the mouse-specific anti-GDAP1 antibody co-precipitate the corresponding human isoform from co-transfected HEK-293T cells. In the negative control, the human GDAP1 is not precipitated with the mouse-specific anti-GDAP1 antibody in the absence of murine GDAP1. (IP: immunoprecipitation, *: IgG heavy chain).

Supplementary Table S1: Assay conditions for spectrophotometric GST-activity assays

Substrate (Company)	Substrate mM	GSH mM	Buffer	Wavelength nm	Extinction coefficient $mM^{-1}cm^{-1}$
1-Chloro-2,4-dinitrobenzene (CDNB) (Sigma)	1	2	0.1 M KPo (pH 6.5)	340	9.6
p-Nitrophenylacetate (*p*NPA) (Sigma)	1	5	KPo (pH 6.5)	400	8.3
p-Nitrobenylchloride (*p*NBC) (Fluka)	0.25	5	KPo (pH 6.5)	310	1.9
Ethacrynic acid (EA) (Sigma)	0.2	0.25	KPo (pH 6.5)	270	5
7-Chloro-4-nitrobenzeno-2-oxa-1,3-diazol (Sigma)	0.2	0.5	NaAc (pH 5.0)	419	14.5
1,2-Epoxy-3-(4-nitrophenoxy)propane (EPNP) (Fisher Scientific)	2	5	KPo (pH 6.5)	360	0.5
Cumene hydroperoxide (Cayman)	n.d.	n.d.	50mM Tris (pH 7.6), 5mM EDTA	340	6.22
t-buthyl hydroperoxide (Fluka)	n.d.	n.d.	50mM Tris (pH 7.6), 5mM EDTA	340	6.22

KPo: potassium phosphate buffer; NaAc: sodium acetate buffer; n.d.: not declared in assay kit
